# The Associations between Immunity-Related Genes and Breast Cancer Prognosis in Korean Women

**DOI:** 10.1371/journal.pone.0103593

**Published:** 2014-07-30

**Authors:** Jaesung Choi, Nan Song, Sohee Han, Seokang Chung, Hyuna Sung, Ji-young Lee, Sunjae Jung, Sue K. Park, Keun-Young Yoo, Wonshik Han, Jong Won Lee, Dong-Young Noh, Daehee Kang, Ji-Yeob Choi

**Affiliations:** 1 Department of Biomedical Sciences, Seoul National University Graduate School, Seoul, Korea; 2 Cancer Research Institute, Seoul National University College of Medicine, Seoul, Korea; 3 Department of Preventive Medicine, Seoul National University College of Medicine, Seoul, Korea; 4 Division of Cancer Epidemiology and Genetics, National Cancer Institute, Bethesda, Maryland, Umited States of America; 5 Cardiovascular Research Institute and Cardiovascular Genome Center, Yonsei University Health System, Seoul, Korea; 6 Department of Surgery, Seoul National University College of Medicine, Seoul, Korea; 7 Department of Surgery, University of Ulsan College of Medicine and ASAN Medical Center, Seoul, Korea; National Cancer Center, Japan

## Abstract

We investigated the role of common genetic variation in immune-related genes on breast cancer disease-free survival (DFS) in Korean women. 107 breast cancer patients of the Seoul Breast Cancer Study (SEBCS) were selected for this study. A total of 2,432 tag single nucleotide polymorphisms (SNPs) in 283 immune-related genes were genotyped with the GoldenGate Oligonucleotide pool assay (OPA). A multivariate Cox-proportional hazard model and polygenic risk score model were used to estimate the effects of SNPs on breast cancer prognosis. Harrell’s C index was calculated to estimate the predictive accuracy of polygenic risk score model. Subsequently, an extended gene set enrichment analysis (GSEA-SNP) was conducted to approximate the biological pathway. In addition, to confirm our results with current evidence, previous studies were systematically reviewed. Sixty-two SNPs were statistically significant at *p*-value less than 0.05. The most significant SNPs were rs1952438 in *SOCS4* gene (hazard ratio (HR) = 11.99, 95% CI = 3.62–39.72, *P* = 4.84E-05), rs2289278 in *TSLP* gene (HR = 4.25, 95% CI = 2.10–8.62, *P* = 5.99E-05) and rs2074724 in *HGF* gene (HR = 4.63, 95% CI = 2.18–9.87, *P* = 7.04E-05). In the polygenic risk score model, the HR of women in the 3^rd^ tertile was 6.78 (95% CI = 1.48–31.06) compared to patients in the 1^st^ tertile of polygenic risk score. Harrell’s C index was 0.813 with total patients and 0.924 in 4-fold cross validation. In the pathway analysis, 18 pathways were significantly associated with breast cancer prognosis (*P*<0.1*)*. The *IL-6R*, *IL-8*, *IL-10RB*, *IL*-*12A*, and *IL*-*12B* was associated with the prognosis of cancer in data of both our study and a previous study. Therefore, our results suggest that genetic polymorphisms in immune-related genes have relevance to breast cancer prognosis among Korean women.

## Introduction

Cancer is a significant health problem in many parts of the worldwide [1], [2]. In Korea, the incidence rate of breast cancer was ranked second and the mortality rate fifth in Korean women, which steadily increased from 1983 to 2010 [3]. The etiology and progression of breast cancer is a multiple-step process caused by combining many factors which involve environmental, hormonal and genetic factors [4], [5]. We focused on genetic factors involved in immune response which was known to play a role in breast cancer prognosis.

The association of immune markers with breast cancer prognosis were well known and the role as key factor of microenvironment of tumor such as tumor suppressor or growth. For example, high density of CD68 which is high-infiltration of tumor-associated macrophages was related with poorer outcome in node-negative breast cancer [6] and CD44 positive patients showed longer overall survival and progression free survival than CD44 negative patients [7]. In addition, cytokines produced by various immune cells were known to modulate the transition from the innate to the adaptive immune response, the activation of anti-tumor cells, persistent oxidative stress, and the angiogenesis of breast cancer [8]–[10]. The prognosis of breast cancer was also known to be associated with single nucleotide polymorphisms (SNPs) in the immune system related genes [11]–[14]. Those reports described that genetic variants of toll-like receptor 4 (*TLR4*), interleukin 12 (*IL-12*), interleukin 2 (*IL-2*), and interleukin 6 (*IL-6*) were related with breast cancer prognosis. However, there have been few studies that investigate the association between comprehensive list of variants in the immunity-related genes and the prognosis of breast cancer.

Given the findings that immune system is related with breast cancer prognosis, we hypothesized that many genetic polymorphisms in immune related genes might be prognostic factor of breast cancer recurrence. In this study, the role of common immune genetic variations to the disease free survival (DFS) of breast cancer was investigated with the multivariate Cox-proportional hazard model by individual variants, polygenic risk score model, and an extended gene set enrichment analysis. Additionally, a systematic review of previous literature that had reported on the associations between variants of the immunity-related genes and the prognosis of various cancers was done.

## Materials and Methods

### Study population

Among subjects of Seoul Breast Cancer Study (SEBCS), a multicenter based case-control study recruiting between 2001 and 2007, the participants in this study were patients diagnosed with histologically confirmed breast cancer in the Seoul National University Hospital during 2002–2004. Based on the sample availability and quality of DNA, 140 breast cancer patients were successfully genotyped [15]. Among them, 107 patients were included in the final analysis after excluding patients without survival status or clinical information or been diagnosed as metastatic breast cancer patients.

During recruitment, well-trained interviewers provided patients with informed consent forms and collected information with a structured questionnaire. Through abstracting the medical chart, information on survival status, hormone receptor status, and TNM stage [16] were obtained.

This study design was approved by the Committee on Human Research of Seoul National University Hospital (IRB No. H-0503-144-004).

### Genotyping

Among 209 samples met the genotyping criteria (concentration >7.5 ng/ul and total amount of DNA >750 ng), 140 cases were successfully genotyped. 283 immune-related candidate genes were composed of 190 innate immune-related genes in innate immune oligonucleotide pool assay (OPA) chip and 93 adaptive immune-related genes in Non-Hodgkin’s lymphoma (NHL) OPA chip as described in previous study [15], [17]. 2,432 Tags SNPs were selected with SNP500 Cancer project database considering the site from 20 kb upstream of the first site of transcription of a candidate gene to 10 kb downstream of the end site of the last exon of the candidate gene and genotyped. Among them, 461 SNPs were excluded from the analysis because of low minor allele frequency (MAF) (<3%) and deviation from Hardy-Weinberg Equilibrium (HWE) (*P<*10^−4^). Finally, a total of 1,971 SNPs in 279 immunity genes were selected for the analysis.

### Statistical method

A DFS was calculated from the date when patients underwent a breast cancer operation to the date of last follow-up or recurrence, such as loco-regional, distant, contralateral recurrence and death from any causes. If patients had no evidence of recurrence, they were censored at the last follow-up date or on June 30, 2011. The median follow-up time was 4.87 years (range, 0.25–6.72 years).

Demographic data including age (<50 and ≥50), body mass index (BMI) (<21.4 and ≥21.4), family history of breast cancer in 1^st^ and 2^nd^ relatives (no and yes), educational level (≤ middle school, high school, and ≥ college or university), smoking status (never and ever), alcohol consumption (never and ever), and menopausal status (premenopausal and postmenopausal), and clinicopathological data including estrogen receptor status (ER) (positive and negative), progesterone receptor status (PR) (positive and negative), and 7^th^ AJCC TNM stage (I, II, and III) were assessed for DFS with the log-rank test and univariate Cox-proportional hazard model. Multivariate Cox-proportional hazard model adjusted for age, ER status, PR status, and TNM stage (I, II, and III) was used to calculate the hazard ratio (HR) and their 95% CI of the effect for each SNP on the DFS of breast cancer based on additive genetic models. If SNPs were located in the same candidate gene and these SNPs had a linkage disequilibrium (LD) (r^2^>0.4), the most significantly associated SNP were selected. To correct the multiple comparison, false discovery rate (FDR) *p*-values were calculated with the Benjamin-Hochberg method [18].

For the polygenic risk score method, the polygenic risk score was calculated by adding the number of risk alleles in each patient based on individual SNP analyses and the patients were categorized into tertiles of polygenic risk score [19]. HR and 95% confidence intervals (CIs) per tertile of polygenic risk score were calculated. After analyzing multivariate Cox-proportional hazard model, Harrell’s C index was calculated to evaluate predictive accuracy of polygenic risk score model [20]. In addition, 4-fold cross-validation method was used to appraise the internal validity of our model; the entire data set was randomly partitioned into 4 equal size subsets. Of the 4 subsets, 3 subsets were used as training data, and a remaining single subset was retained as the validation data for testing the model. Significantly associated SNPs with prognosis of breast cancer were firstly estimated in training set and then Harrell’s C index was estimated based on those SNPs in validation set. The cross-validation process was then repeated 4 times. The summary of these 4 Harrell’s indices was assessed by fixed-effect model meta-analysis.

The GSEA-SNP method was used to reveal the biological function of the SNPs which were significantly related to breast cancer prognosis [21]. Pathway information was obtained from the Molecular Signatures Database (MSigDB) which collected annotated gene sets from the following online databases; BioCarta, KEGG, Pathway Interaction Database, Reactome, SigmaAldrich, Signaling Gateway, Signal Transduction KE, and SuperArray. In addition, gene sets that have been extracted from experimental studies were included in the database. The curated gene sets were downloaded from MSigDB (version 4.0, C2). Because there was a chance of the biological pathway being narrowly defined, each pathway was set up to contain at least three genes in the following analyses. The names of gene sets were described with ‘brief description’ rather than ‘standard name’ which is available on the GSEA web (http://www.broadinstitute.org/gsea/index.jsp), because standard name equivocally explained function of gene set.

The statistical significance of the effects was estimated with a *p-*value less than 0.05 in both multivariate Cox-proportional hazard model by individual variants and polygenic risk score models and 0.1 in GSEA-SNP. The SAS statistical software package version 9.3, PLINK program version 1.07, and R 2.15.1 packages (GenABEL), STATA statistical software version 12.0 were used for the analyses.

### Systematic review

Previous studies conducting analyses to find associations between immunity-related genetic factors and the prognosis of cancer in the epidemiologic field were selected for Jan 2000 through Dec 2013 (Figure 2). Available studies for systematic review were searched in the PubMed and EMBASE database with a set of keywords that delineated breast cancer as well as other cancers, immune, genetic factors, and survival; cancer AND immune AND polymorphism AND survival. Abstracts were reviewed to identify reports examining associations between immunity-related genetic factors and clinical outcomes including recurrence and death. Literatures were excluded in the following circumstances; review paper, studies unrelated with genomic epidemiology, using SNPs located in non-immune related genes, duplicated in both databases, with no survival or recurrence data reported for survival analysis and no hazard ratios (HRs) reported which were estimated with the Cox-proportional hazard model for the associations of immunity-related genetic factors with cancer outcomes (Figure 2). In cases of duplication between both databases, the studies were deemed to have been searched in the PubMed database. The following data were extracted from each eligible study from the literature; disease site, authors, genes assessed, number of polymorphisms assessed, number of patients and events including recurrence, death, follow-up period, type of outcome, and covariates. Associations between polymorphisms and the outcome of each cancer were recorded as HR with 95% CI and adjustments. Because different nomenclatures and names for polymorphisms were used in the studies, all polymorphisms were named by RefSNP (rs) numbers. We followed the Preferred Reporting Items for Systematic Review and Meta-Analysis (PRISMA) statement and checklist as a methodological template for this review (Table S1).

## Results

Table 1 shows the characteristics of the 107 patients including 20 patients who had the events. Among the 107 cases, BMI, PR status, and TNM stage showed a significant association with the prognosis on the DFS of breast cancer (*P*<0.05, log-rank test), while there were no significant differences in age, family history of breast cancer, educational level, menopausal status, smoking status, alcohol consumption, and ER status.

**Table 1:** Characteristic of study participants.

Characteristics	No. of patients (%)	No. of events (%)	*P* a	HR^b^	(95% CI)	*P* b
Total	107 (100.0)	20 (100.0)				
Age (Mean ± SD)	50.6±8.2	52.5±10.6	0.60			
<50	54 (50.5)	11 (55.0)		1.00		
≥50	53 (49.5)	9 (45.0)		0.79	(0.33–1.91)	0.60
Body mass index (Mean ± SD)	23.7±2.9	24.4±2.13	<0.02			
<21.4 (median)	30 (33.3)	1 (5.0)		1.00		
≥21.4	77 (66.7)	19 (95.0)		7.30	(0.98–54.61)	0.05
Family history			1.00			
No	97 (90.7)	18 (90.0)		1.00		
Yes	10 (9.3)	2 (10.0)		1.00	(0.23–4.37)	1.00
Educational level			0.46			
≤Middle school	30 (28.3)	4 (20.0)		1.00		
High school	46 (43.4)	11 (55.0)		1.95	(0.62–6.13)	0.26
≥College or university	30 (28.3)	5 (25.0)		1.27	(0.34–4.73)	0.72
Menopausal status			0.71			
Premenopausal	62 (58.5)	11 (55.0)		1.00		
Postmenopausal	44 (41.5)	9 (45.0)		1.18	(0.49–2.84)	0.72
Smoking status			0.10			
Never	100 (93.5)	17 (85.0)				
Ever	7 (6.5)	3 (15.0)		2.70	(0.78–9.17)	0.12
Alcohol consumption			0.66			
Never	70 (65.4)	14 (70.0)				
Ever	37 (34.6)	6 (30.0)		0.81	(0.31–2.10)	0.66
Estrogen receptor status			0.07			
Positive	66 (62.3)	9 (45.0)		1.00		
Negative	40 (37.7)	11 (55.0)		2.19	(0.90–5.28)	0.08
Progesterone receptor status			0.01			
Positive	53 (50.5)	5 (25.0)		1.00		
Negative	52 (49.5)	15 (75.0)		3.39	(1.23–9.37)	0.02
TNM stage			<0.01			
0/I	48 (45.3)	4 (20.0)		1.00		
II	40 (37.7)	7 (35.0)		2.20	(0.64–7.56)	0.21
III	18 (17.0)	9 (50.0)		8.54	(2.62–27.88)	<0.01

a Log rank test.

b Univariate Cox-proportional hazard model.

The associations of immunity-related genetic factors on DFS of breast cancer prognosis are presented in Table 2. Among 1,971 SNPs, 80 SNPs were significantly associated with the DFS of breast cancer. The 62 SNPs were remained after excluding those with high LD (r^2^>0.4) and 3 SNPs were still significant at FDR p-value less than 0.05. The SNPs were rs1952438 in *SOCS4* gene (HR = 11.99, 95% CI = 3.62–39.72, *P* = 4.84E-05), rs2289278 in *TSLP* gene (HR = 4.25, 95% CI = 2.10–8.62, *P* = 5.99E-05) and rs2074724 in *HGF* gene (HR = 4.63, 95% CI = 2.18–9.87, *P* = 7.04E-05).

**Table 2:** Associations between the genetic variations of immunity-related genes and breast cancer disease free survival in the additive model (significance level, *P*<5.00E-02).

Gene	Location	SNP	MAF	HR^a^	(95% CI)	*P*
*SOCS4*	intronic	rs1952438	0.04	11.99	(3.62–39.72)	4.84E-05
*TSLP*	UTR5	rs2289278	0.15	4.25	(2.10–8.62)	5.99E-05
*HGF*	intronic	rs2074724	0.11	4.63	(2.18–9.87)	7.04E-05
*IL-17C*	intronic	rs2254073	0.15	4.24	(1.90–9.49)	4.31E-04
*BCL2*	intergenic	rs9989529	0.19	3.80	(1.63–8.84)	1.98E-03
*CCL2*	intergenic	rs17652343	0.08	4.57	(1.74–11.97)	2.01E-03
*ITGB2*	intronic	rs2838727	0.04	6.57	(1.84–23.44)	3.70E-03
*TRAF2*	intergenic	rs908831	0.14	3.79	(1.54–9.36)	3.79E-03
*NBN*	downstream	rs2142097	0.42	3.55	(1.48–8.49)	4.40E-03
*SELE*	intergenic	rs4656701	0.35	0.28	(0.11–0.71)	7.41E-03
*CCR1*	downstream	rs3136671	0.19	3.05	(1.33–7.00)	8.47E-03
*HGF*	intronic	rs5745752	0.33	0.29	(0.11–0.73)	9.22E-03
*IL-12A*	intergenic	rs9811792	0.31	0.23	(0.08–0.71)	1.01E-02
*MIF*	ncRNA_exonic	rs1007888	0.41	2.39	(1.22–4.67)	1.11E-02
*ITGB2-AS1*	ncRNA_exonic	rs2070946	0.12	2.98	(1.28–6.93)	1.11E-02
*MIF*	ncRNA_intronic	rs2000466	0.18	3.37	(1.32–8.60)	1.12E-02
*ALOXE3*	intronic	rs3027215	0.07	3.17	(1.28–7.87)	1.27E-02
*IFNAR2*	intronic	rs2073362	0.15	3.86	(1.33–11.17)	1.28E-02
*XDH*	intergenic	rs10490361	0.46	0.44	(0.23–0.84)	1.35E-02
*CCL8*	intergenic	rs3138034	0.07	3.59	(1.29–9.96)	1.42E-02
*SOCS2*	intronic	rs3782415	0.48	2.38	(1.18–4.83)	1.60E-02
*DEF6*	intronic	rs6938946	0.34	2.26	(1.16–4.39)	1.68E-02
*ABHD16A*	intronic	rs2295663	0.10	2.55	(1.16–5.59)	1.93E-02
*LBP*	intronic	rs12624843	0.30	0.33	(0.13–0.84)	2.03E-02
*IL-18*	intergenic	rs243908	0.33	3.59	(1.22–10.61)	2.05E-02
*IL-10RB*	UTR3	rs1058867	0.32	2.62	(1.14–6.04)	2.33E-02
*IL-6R*	intergenic	rs11265608	0.04	4.15	(1.21–14.21)	2.36E-02
*IRAK4*	intronic	rs4251460	0.11	2.78	(1.15–6.73)	2.38E-02
*TRAF5*	intronic	rs6684874	0.29	0.29	(0.10–0.85)	2.46E-02
*MIF*	ncRNA_intronic	rs17004044	0.17	0.23	(0.06–0.83)	2.48E-02
*XDH*	intronic	rs1429372	0.38	0.43	(0.20–0.91)	2.70E-02
*LMAN1*	intronic	rs12953981	0.41	0.41	(0.19–0.91)	2.74E-02
*ALOXE3*	intronic	rs3027208	0.43	0.44	(0.21–0.91)	2.76E-02
*CCL11*	intergenic	rs4795904	0.08	3.11	(1.13–8.56)	2.81E-02
*IL-12B*	intergenic	rs4921468	0.22	2.54	(1.10–5.87)	2.85E-02
*IL-4R*	UTR3	rs8832	0.42	0.39	(0.17–0.91)	2.85E-02
*IL-12A*	intergenic	rs747825	0.15	0.10	(0.01–0.79)	2.90E-02
*SCNN1A*	intronic	rs3759324	0.36	2.10	(1.07–4.14)	3.03E-02
*ITGB2*	intronic	rs1474552	0.23	0.26	(0.08–0.88)	3.06E-02
*C6*	intronic	rs13168926	0.40	0.40	(0.18–0.92)	3.08E-02
*FGF2*	intergenic	rs308447	0.08	2.89	(1.09–7.65)	3.25E-02
*IL-10*	intronic	rs3021094	0.42	0.40	(0.17–0.93)	3.26E-02
*SELE*	intergenic	rs4656699	0.20	0.31	(0.11–0.92)	3.41E-02
*STK19*	intronic	rs389883	0.26	1.96	(1.05–3.67)	3.46E-02
*STAT4*	intronic	rs1031509	0.31	0.43	(0.19–0.94)	3.53E-02
*NCF4*	intronic	rs2075938	0.39	2.17	(1.05–4.51)	3.66E-02
*SLC2A11*	intergenic	rs1984309	0.39	0.44	(0.20–0.95)	3.68E-02
*BPI*	intronic	rs2275954	0.40	2.19	(1.05–4.59)	3.70E-02
*TNFRSF1A*	intronic	rs4149577	0.41	0.37	(0.14–0.94)	3.70E-02
*KLK15*	upstream	rs3745523	0.29	2.07	(1.04–4.12)	3.81E-02
*BCL2*	intronic	rs12458289	0.28	2.27	(1.04–4.96)	4.00E-02
*MBL2*	intergenic	rs11003134	0.20	8.09	(1.08–60.37)	4.16E-02
*BCL10*	intergenic	rs6693365	0.30	2.36	(1.03–5.39)	4.18E-02
*SELE*	intronic	rs3917412	0.28	2.13	(1.03–4.40)	4.21E-02
*CD180*	intergenic	rs6890674	0.15	2.27	(1.03–5.02)	4.29E-02
*MAL*	intronic	rs3113002	0.35	0.45	(0.21–0.98)	4.30E-02
*AICDA*	UTR3	rs11046349	0.12	2.81	(1.03–7.69)	4.44E-02
*C1QA*	intronic	rs2935542	0.14	2.29	(1.02–5.13)	4.49E-02
*IRF4*	intergenic	rs11242867	0.29	2.16	(1.02–4.61)	4.50E-02
*IL-8*	intergenic	rs4694178	0.40	0.48	(0.23–0.99)	4.61E-02
*MASP1*	intronic	rs3105782	0.15	2.30	(1.01–5.24)	4.70E-02
*MUC2*	intergenic	rs4077757	0.03	3.88	(1.01–14.90)	4.80E-02

a Multivariate Cox proportional hazard model adjusted for age, estrogen receptor status, progesterone receptor status and TNM stage.

**Figure 1:**
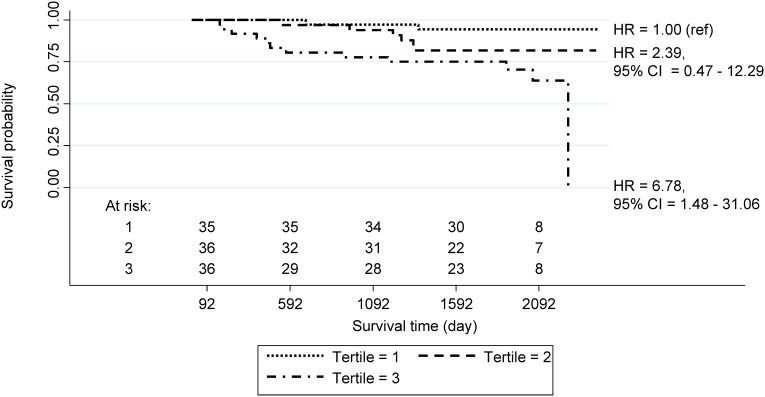
Associations of the polygenic risk score on breast cancer disease free survival. Kaplan-Meier survival curve and estimated hazard ratios (HRs) of breast cancer in groups defined by tertile derived from the polygenic risk scores of the 107 patients with all 62 SNPs.

Figure 1 presents the Kaplan-Meier survival curve and estimated HRs of breast cancer in groups defined by tertile derived from the polygenic risk scores of the 107 patients with all 62 SNPs. The HR was significantly increased as the score increased (*p* for trend = 0.01). The HR of women in the 3^rd^ tertile was 6.78 (95% CI = 1.48–31.06) compared to patients in the 1^st^ tertile of polygenic risk score. Table 3 shows the predictive accuracy and validation results of polygenic risk score model. The Harrell’s C index of total patients is 0.813, and summarized Harrell’s C index of cross validation is 0.924.

**Figure 2:**
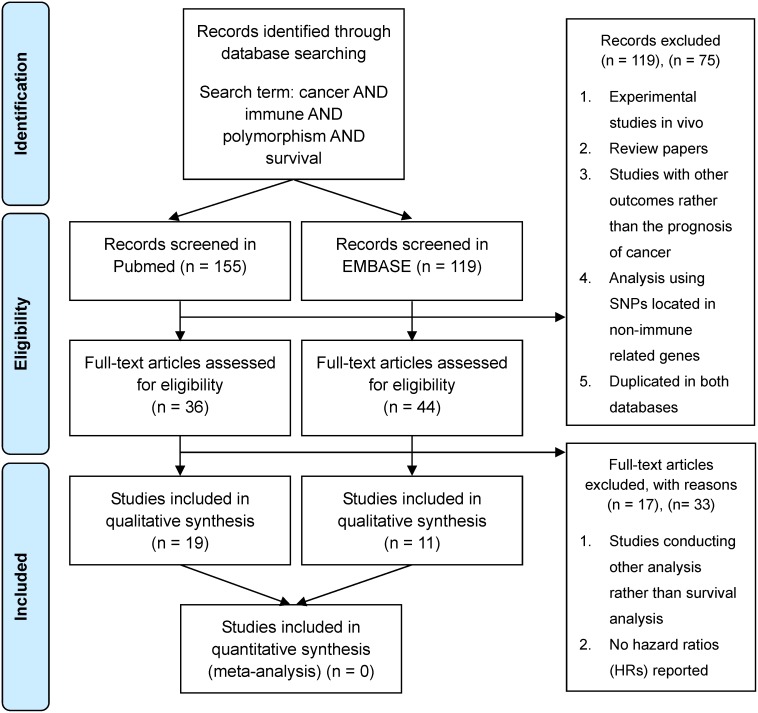
Overview of inclusion and exclusion criteria in systematic review.

**Table 3:** Harrell’s C index for polygenic risk score estimated by 4-fold cross-validation.

Group	No. of SNPs in CV set	Harrell’s C index	Standard error	(95% CI)
All		0.813	0.48	(0.72–0.91)
CV set1	25	0.885	0.09	(0.70–1.07)
CV set2	40	0.910	0.06	(0.78–1.04)
CV set3	32	0.940	0.03	(0.88–1.00)
CV set4	36	0.909	0.04	(0.82–1.00)
Summary^a^		0.924	0.02	(0.88–0.97)

a The summary of Harrell’s C index for 4 test sets calculated by fixed-effect meta-analysis.

In GSEA-SNP analysis, our results showed that 18 pathways with 62 SNPs in 56 immunity-related genes had significant association with the DFS of breast cancer at a *p*-value less than 0.1 (Table 4); set ‘Myc targets1’: targets of c-Myc identified by ChIP on chip in cultured cell lines, focusing on E-box-containing genes; high affinity bound subset (including *BCL2* and *NBN*, *P* = 0.04), mitochondrial genes; based on literature and sequence annotation resources and converted to Affymetrix HG-U133A probe sets (including *BCL2* and *NBN*, *P* = 0.04), genes down-regulated in T24 (bladder cancer) cells in response to the photodynamic therapy (PDT) stress (including *BCL2* and *CCL2*, *P* = 0.04), genes transiently induced only by the second pulse of *EGF* in 184A1 cells (mammary epithelium) (including *IRF3*, *TRAF5*, *KLK15* and *IL5R*, *P* = 0.02).

**Table 4:** Pathway analysis for immune related genes on breast cancer disease free survival using GSEA-SNP method (*P*<0.1).

Included genes (No. of SNPs)	HR^a^	(95% CI)	Enrichment Score	Normal *P* b	Gene set (pathway)	Reference	
*BCL2* (2), *NBN* (1)	2.65	(1.69–4.14)	0.8594	0.04	Set ‘Myc targets1’: targets of c-Myc identified byChIP on chip in cultured cell lines, focusing onE-box-containing genes; high affinity bound subset	Benporath *et al.*	[36]
					Mitochondrial genes; based on literature and sequenceannotation resources and converted to AffymetrixHG-U133A probe sets	Mootha *et al.*	[43]
*BCL2* (2), *CCL2* (1)	3.12	(1.98–4.90)	0.8438	0.04	Genes down-regulated in T24 (bladder cancer) cells inresponse to the photodynamic therapy (PDT) stress	Buytaert *et al.*	[29]
*TSLP* (1), *BCL* (2), *TRAF5* (1), *MASP1* (1)	2.29	(1.59–3.32)	0.7392	0.06	Genes down-regulated in prostate cancer samples	Liu *et al.*	[44]
*SOCS4* (1), *HGF* (2)	2.39	(1.73–3.29)	0.8552	0.07	Human environmental stress response genes notchanged in primary fibroblasts from Wilmorsyndrom (WS) patients in response to 4NQO treatment	Kyng *et al.*	[35]
					Human environmental stress response genes notchanged in primary fibroblasts from old donors inresponse to UV radiation	Kyng *et al.*	[35]
*TSLP* (1), *ALOXE3* (2), *BCL2* (2), *MAL* (1), *IRF4* (1)	1.94	(1.49–2.52)	0.7163	0.08	Set ‘H3K27 bound’: genes posessing the trimethylatedH3K27 (H3K27me3) mark in their promoters inhuman embryonic stem cells, as identified by ChIPon chip.	Benporath *et al.*	[36]
*TSLP* (1), *ALOXE3* (2), *BCL2* (2), *MAL* (1)	1.99	(1.52–2.62)	0.7393	0.08	Set ‘Suz12 targets’: genes identified by ChIP onchip as targets of the Polycomb protein *SUZ12* inhuman embryonic stem cells.	Benporath *et al.*	[36]
*HGF* (2), *BCL2* (2)	2.48	(1.71–3.60)	0.7734	0.09	Focal adhesion	KEGG	[45]
			0.7734	0.09	Direct p53 effectors	PID	[46]
*BCL2* (2), *LBP* (1)	2.57	(1.66–3.97)	0.8136	0.09	Genes in the expression cluster ‘Early ProgenitorsShared’: up-regulated in hematopoietic progenitorsfrom adult bone marrow and from fetal liver.	Ivanova *et al.*	[47]
*TSLP* (1), *BCL2* (2), *MAL* (1)	2.14	(1.58–2.88)	0.7617	0.10	Set ‘EED targets’: genes identified by ChIP on chip as targets of the Polycomb protein *EED* in human embryonic stem cells.	Benporath *et al.*	[36]
			0.7617	0.10	Set ‘PRC2 targets’: Polycomb Repression Complex 2(PRC) targets; identified by ChIP on chip on humanembryonic stem cells as genes that: posess thetrimethylated H3K27 mark in their promoters and are bound by *SUZ12* and *EED* Polycomb proteins.	Benporath *et al.*	[36]
*IRF3* (1), *TRAF5* (1), *KLK15* (1), *IL4R* (1)	0.75	(0.48–1.16)	−0.7414	0.02	Genes transiently induced only by the second pulse of*EGF* in 184A1 cells (mammary epithelium).	Zwang *et al.*	[48]
*MBL2* (1), *MASP1* (1), *C6* (1)	2.80	(1.53–5.14)	−0.8438	0.06	Lectin Induced Complement Pathway	Biocarta	[49]
			−0.8438	0.06	Genes down-regulated in liver samples of liver-specificknockout of *HNF4A*	Ohguchi *et al.*	[50]
*IRF4* (1), *TRAF5* (1), *MUC* (1)	0.56	(0.24–1.29)	−0.8136	0.07	Genes up-regulated in the HMEC cells (primarymammary epithelium) upon expression of *TP53* offadenoviral vector.	Perez *et al.*	[51]
*CCR1* (1), *IL8* (1), *TNFRSF1A* (1)	0.72	(0.43–1.20)	−0.7188	0.09	Genes up-regulated in circulating endothelial cells(CEC) from cancer patients compared to those fromhealthy donors	Smirrnov *et al.*	[52]

aMutivariate Cox proportional hazard model adjusted for age, hormone status and TNM stage according to polygenic risk score estimated by using SNPs included in each pathway.

b *P* value for GSEA-SNP analysis.

Table 5 showed 30 studies resulted from systematic review for survival analyses estimating effects of immune-related genetic factors on various cancers. In the studies, eighty eight SNPs in 58 immunity genes were significantly associated with the prognosis of cancer patients (Table 6). In those results, there were 29 genes overlapped in both our study and previous studies, but no SNPs overlapped. Among them, *IL-6R*, *IL-8*, *IL-10RB*, *IL*-*12A*, and *IL*-*12B* was significantly associated with the prognosis of cancer consistent to our finding.

**Table 5:** Characteristics of previous studies.

Types of cancer	Study authors	Genes assessed	No. of SNPs assessed	No. of patients	No. of events	Follow-up period, yrs	Types of outcome^j^	Adjusted covariates^k^	Ref
Breast	Yang *et al*.	*TLR4*	4	604	-	4.9	OS	-	[11]
	You *et al*.	*IL-21*	4	891	121	5.0	OS	age, age at menarche (years), menstrual status, BMI, pathological type, stage, ER status, PR status, family history of any cancer	[12]
	Hu *et al*.	*IL-2*	2	638	-	5.0	OS	-	[13]
	DeMichele *et al*.	*IL-6*	4	346	-	11.2	DFS	age at diagnosis, race, *CYP3A4, GSTM1*	[14]
	Bewick *et al.*	*ERCC1* and *ERCC2*	3	95	91	0.9 (PFS) 1.9 (BCSS)	PFS, BCSS	age	[53]
Colorectal	Lu *et al*.	*REG4, BML,* and *CD209*	15	414	203	4.7	OS	age at diagnosis, gender, TNM stage.	[54]
	Castro *et al*.	13 immune genes	19	582	150	13.0	OS	age at diagnosis, T, N stage.	[55]
	Slattery *et al*.	11 immune genes	50	1555^a^	309	>5.0	OS	age, study center, ethnic group/ethnicity, sex, TNM stage, tumor molecular phenotype	[56]
				754^b^	171	>5.0	OS		
	Bondurant *et al*.	13 immune genes	59	1956^a^	309	>5.0	OS	age, study center, ethnic group/ethnicity, sex, AJCC stage and tumor molecular phenotype	[57]
				954^b^	171	>5.0	OS		
Non-small cell lung	Bi *et al*.	*Cox-2*	5	136	-	5.0	OS	age, sex, smoking status, KPS, weight loss, histology, clinical stages, chemotherapy, radiation dosage	[58]
	Dai *et al*.	52 immune genes	178	568	311	6.0	OS	smoking status, histology, stage, surgical operation, chemotherapy, or radiotherapy status	[59]
	Sung *et al*.	*FasL*	1	385	124	2.6^i^	OS, RFS	age, gender, smoking, tumor type, stage	[60]
	Yuan *et al.*	*TGF-β1*	3	205	-	1.4^i^	OS, DMFS	age, sex, race, KPS, smoking status, tumor histology, gross tumor volume, disease stage, receipt of chemotherapy or concurrent radiochemotherapy, number of cycles of chemotherapy, and radiation dose received	[61]
	Xue *et al.*	*TGF-β1*	2	109	85	1.2^i^	OS	age, gender, smoking status, histology, stage, radiation technique, radiation dose, and chemotherapy	[62]
	Schabath *et al.*	53 inflammation-related genes	326	651	-	2.1^i^	OS	age, gender, race, smoking status, stage, histology and first-course treatment.	[63]
	Guan *et al.*	*TNF-α* and *TNFRSF1B*	5	225	155	1.9	OS	age, gender, ethnicity, smoking status, tumor histology, KPS, tumor stage, node status, application of chemotherapy and radiotherapy dose	[64]
	Pine *et al.*	*MBL2*	5	558 (white population)	405	3.8	OS	sex, stage (III–IV versus I-II), age at diagnosis, current smoking status, and pack-years of smoking	[65]
Bladder	Guirado *et al*.	*C13ORF31, NOD2, TLR10,* and *RIPK2*	5	349	66	3.9^i^	OS	-	[66]
Renal cell carcinoma	Schutz *et al*.	70 immune genes	290	403^c^	184	5.3	RFS	ECOG performance status, clinical stage, tumour size, tumour Fuhrman grade, histology (clear cell *vs* non clear cell)	[67]
				151^c^	44	8.8	RFS		
Lymphoma	Aschebro-okkilfoy *et al*.	40 immune genes	82	496	211	12.0	OS	age, education, stage, B-symptom, initial treatment.	[68]
	Charbonneau *et al*.	30 immune genes	167	107^d^	60	8.3	EFS	clinical risk score, which accounts for the effects of treatment type and FLIPI (FL) or IPI (DLBCL)	[69]
				82^e^	39	8.3	EFS		
	Habermann *et al*.	44 immune genes	73	365	96	4.8	OS	age and clinical and demographic factors.	[70]
	Cerhan *et al*.	44 immune genes	73	278	59	4.8	OS	age, clinical, demographic factors	[42]
Melanoma	Lenci *et al*.	15 type *IFN* genes	44	625	174	-	OS, DFS	gender, age and Breslow thickness	[71]
Ovarian	Goode *et al*.	54 immune genes	1536	3665	1529	5.4	OS	study site, tumor stage, race, tumorgrade	[72]
Pancreatic	Reid-Lombardo *et al.*	102 inflammatory genes	1536	400^f^	318	2.0^i^	OS	age, sex, body mass index class, stage, margin status (R0, R1, R2), grade, tumor size, and lymph node status	[73]
				443^g^	420	0.8^i^	OS		
				465^h^	454	0.6^i^	OS		
Osteosarcoma	Biason *et al.*	*XPD*, *XPG*, and *XPA*	5	130	57	3.0	EFS	covariate which were significant in the univariate analysis	[74]
Esophageal	Lee *et al.*	*ERCC2* and *ERCC4*	2	400	310	-	OS, PFS	T stage, N stage, Cell type, esophagectomy, CCRT	[75]
Head and neck	Lundberg *et al.*	*TGF-β1*	1	34	14	4.0	OS, DFS	age, sex, cisplatin dose (mg/m2), RT dose (Gy) and treatment modality	[76]
Myeloma	Vangsted *et al.*	*IL-1β, IL-6, IL-10, PPARγ2,* and *COX-2*	6	348	68	-	OS	*β*2-microglobulin, creatinine and Durie–Salmon stage	[77]

a Colon cancer patients, ^b^Rectal cancer patients, ^c^403 cases are discovery cohort and 151 cases are validation cohort, each cohort selected from different center, ^d^Follicular lymphoma patients, ^e^Diffuse large B-cell lymphoma patients, ^f^Patients who had undergone pancreatic resection operation, ^g^Patients whose cancer locally advanced, ^h^Patients whose cancer had metastasized, ^i^Median survival time, ^j^OS, overall survival; DFS, disease free survival; RFS, relapse free survival; EFS, event free survival; DMFS, distant metastasis-free survival; BCSS, breast cancer specific survival, ^k^AJCC, american joint committee on cancer; KPS, karnofski performance status; ECOG, eastern cooperative oncology group; CCRT, concurrent neoadjuvant chemoradiotherapy; FIGO, international federation of gynecology and obstetric.

**Table 6:** Genes that have significant SNPs of each study in the review of previous studies.

Gene	SNP	Primary endpoint^a^	HR	(95% CI)	*P*	Type of cancer^b^	Ref
*C7*	rs324058	EFS	1.66	(0.87–3.17)	0.04	Lymphoma	[69]
*C9*	rs1421094	EFS	0.54	(0.32–0.90)	0.02	Lymphoma	[69]
*CCR5*	rs1800940	OS	0.73	(0.53–1.00)	-	Lymphoma	[68]
*CD46*	rs2466571	EFS	1.49	(0.86–2.61)	0.05	Lymphoma	[69]
*CD55*	rs2564978	EFS	0.52	(0.30–0.88)	<0.01	Lymphoma	[69]
*CD80*	rs13071247	OS	1.73	(1.26–2.39)	<0.01	Ovarian cancer	[72]
	rs7804190	OS	1.14	(1.06–1.23)	<0.01	Ovarian cancer	[72]
*CFH*	rs3766404	EFS	2.25	(1.31–3.87)	<0.01	Lymphoma	[69]
	rs1329423	EFS	0.49	(0.29–0.38)	<0.01	Lymphoma	[69]
*CFHR1*	rs436719	EFS	0.57	(0.34–0.96)	0.03	Lymphoma	[69]
*CFHR5*	rs6694672	EFS	2.63	(1.41–4.92)	<0.01	Lymphoma	[69]
*CLU*	rs3087554	EFS	0.46	(0.21–1.00)	0.05	Lymphoma	[69]
*COX-2*	rs689466	OS	0.58	(0.39–0.86)	0.01	NSCLC	[58]
*ERCC2*	rs238406	OS	1.64	(1.08–2.50)	0.02	Esophageal cancer	[75]
	rs238406	PFS	1.76	(1.17–2.66)	0.01	Esophageal cancer	[75]
	rs1799793	BCSS	1.90	(1.06–3.26)	0.04	Breast cancer	[53]
	rs1799793	EFS	0.23	(0.05–0.99)	0.01	Osteosarcoma	[74]
*FasL*	rs763110	OS	1.46	(1.13–1.87)	<0.01	NSCLC	[60]
	rs763110	RFS	1.71	(1.33–2.21)	<0.01	NSCLC	[60]
*GATA3*	rs10905278	OS	1.82	(1.31–2.53)	<0.01	Pancreatic cancer	[73]
*IFNAR1*	rs2257167	EFS	0.74	(0.55–1.00)	0.05	NSCLC	[63]
*IFNGR1*	rs1327474	OS	0.69	(0.50–0.94)	0.02	Colorectal cancer	[56]
	rs9376267	OS	1.37	(1.09–1.73)	0.01	Colorectal cancer	[56]
*IFNGR2*	rs2834211	OS	1.32	(1.01–1.72)	0.04	Colorectal cancer	[56]
	rs2834213	OS	2.04	(1.16–3.57)	0.01	Colorectal cancer	[56]
*IFNW1*	rs10964859	OS	1.80	(1.02–3.16)	0.04	Melanoma	[71]
*IL-10RB*	rs8128184	EFS	1.59	(1.11–2.29)	0.01	NSCLC	[63]
*IL-12A*	rs2243148	EFS	1.28	(1.03–1.58)	0.03	NSCLC	[63]
*IL-12B*	rs3212227	OS	1.83	(1.09–3.06)	<0.01	Lymphoma	[42]
*IL-13*	rs1295683	EFS	1.39	(1.03–1.87)	0.03	NSCLC	[63]
*IL-1A*	rs3783546	OS	2.07	(1.28–3.36)	0.02	Colorectal cancer	[57]
	rs1800587	OS	1.90	(1.26–2.87)	<0.01	Lymphoma	[70]
*IL-1B*	rs1143623	OS	1.37	(1.09–1.72)	0.01	Colorectal cancer	[57]
	rs1143627	OS	0.50	(0.30–1.00)	0.04	Myeloma	[77]
*IL-1RN*	rs454078	OS	1.93	(1.11–3.34)	0.03	Lymphoma	[42]
*IL-2*	rs2069763	OS	1.43	(1.15–3.82)	-	Breast cancer	[13]
	rs2069762	OS	1.80	(1.06–3.05)	0.01	Lymphoma	[42]
*IL-21*	rs12508721	OS	0.45	(0.30–0.67)	<0.01	Breast cancer	[12]
*IL-23R*	rs6682925	OS	1.34	(1.05–1.70)	-	NSCLC	[59]
*IL-3*	rs181781	OS	2.47	(1.11–5.53)	0.03	Colorectal cancer	[57]
*IL-5*	rs2069807	OS	4.56	(1.98–10.5)	<0.01	Lymphoma	[70]
	rs2069818	OS	5.58	(1.66–18.6)	0.01	Lymphoma	[42]
*IL-5R*	rs11713419	OS	6.60	(2.42–18.02)	-	NSCLC	[59]
*IL-6*	rs1800796	OS	0.42	(0.23–0.77)	-	Lymphoma	[68]
	rs1800797	DFS	1.60	(1.09–2.35)	0.02	Breast cancer	[14]
*IL-6R*	rs4240872	EFS	0.75	(0.59–0.95)	0.02	NSCLC	[63]
*IL-8*	rs4073	OS	2.14	(1.26–3.63)	-	Lymphoma	[42]
	rs2227307	OS	1.90	(1.12–3.22)	-	Lymphoma	[42]
	rs2227306	OS	1.96	(1.07–3.28)	-	Lymphoma	[42]
	rs12506479	EFS	1.32	(1.08–1.62)	0.01	NSCLC	[63]
*IL-8RB*	rs1126579	OS	1.61	(1.05–2.46)	0.02	Colorectal cancer	[57]
	rs1126580	OS	2.11	(1.28–3.50)	<0.01	Lymphoma	[70]
*IRF2*	rs12504466	OS	1.51	(1.14–1.99)	<0.01	Colorectal cancer	[56]
	rs13116389	OS	1.38	(1.09–1.75)	0.01	Colorectal cancer	[56]
	rs2797507	OS	0.77	(0.61–0.98)	0.03	Colorectal cancer	[56]
	rs3775582	OS	0.67	(0.50–0.89)	0.01	Colorectal cancer	[56]
	rs7655800	OS	1.33	(1.04–1.70)	0.02	Colorectal cancer	[56]
	rs793801	OS	1.39	(1.01–1.91)	0.04	Colorectal cancer	[56]
	rs1425551	OS	1.50	(1.03–2.18)	0.04	Colorectal cancer	[56]
	rs3756094	OS	0.36	(0.20–0.66)	<0.01	Colorectal cancer	[56]
	rs3822118	OS	1.47	(1.08–2.01)	0.02	Colorectal cancer	[56]
	rs807684	OS	0.30	(0.14–0.66)	<0.01	Colorectal cancer	[56]
	rs1044873	OS	1.32	(1.04–1.68)	0.03	Colorectal cancer	[56]
	rs305083	OS	1.31	(1.04–1.65)	0.02	Colorectal cancer	[56]
*IRF6*	rs2013196	OS	1.29	(1.02–1.63)	0.03	Colorectal cancer	[56]
*LRRC32*	rs3781699	OS	2.32	(1.45–3.71)	<0.01	Ovarian cancer	[72]
	rs7944357	OS	2.04	(1.34–3.10)	<0.01	Ovarian cancer	[72]
*MBL2*	rs7096206	OS	0.55	(0.42–0.73)	<0.01	NSCLC	[65]
*MET*	rs11762213	RFS	1.86	(1.17–2.95)	0.01	Renal cell cancer	[67]
*NFKB*	rs7157810	OS	1.43	(1.16–1.75)	<0.01	Pancreatic cancer	[73]
*NOD2*	rs9302752	OS	3.19	(2.04–4.34)	-	Bladder cancer	[66]
*NOS3*	rs1799983	OS	1.39	(1.14–1.70)	<0.01	Pancreatic cancer	[73]
*REG4*	rs2994809	DFS	2.00	(1.18–3.39)	0.01	Colorectal cancer	[54]
	rs2994811	OS	1.35	(1.02–1.78)	0.03	Colorectal cancer	[54]
*RGS1*	rs10921202	OS	2.93	(1.77–4.84)	<0.01	Ovarian cancer	[72]
*RIPK1*	rs2326173	OS	1.44	(1.20–1.74)	<0.01	Pancreatic cancer	[73]
*SOCS3*	rs8064821	OS	0.65	(0.49–0.87)	<0.01	Pancreatic cancer	[73]
*STAT1*	rs12693591	OS	0.68	(0.55–0.86)	<0.01	Pancreatic cancer	[73]
*TGF-β1*	rs10469	OS	1.46	(1.01–2.11)	0.04	NSCLC	[61]
	rs1982073	DMFS	1.59	(1.01–2.50)	0.05	NSCLC	[61]
	rs1982073	DFS	3.23	(1.19–8.77)	0.02	HNSCC	[76]
	rs1800469	OS	0.46	(0.25–0.87)	0.02	NSCLC	[62]
*TGFBR1*	rs10512263	EFS	0.59	(0.37–0.94)	0.03	NSCLC	[63]
	rs868	EFS	1.28	(1.01–1.61)	0.04	NSCLC	[63]
*TGFBR2*	rs2043136	EFS	0.74	(0.58–0.95)	0.02	NSCLC	[63]
*TLR1*	rs5743551	OS	0.78	(0.62–0.97)	-	NSCLC	[59]
*TLR10*	rs4129009	OS	0.49	(0.18–0.80)	-	Bladder cancer	[66]
*TLR3*	rs3775291	OS	1.93	(1.14–3.28)	-	Colorectal cancer	[55]
	rs3775291	OS	1.37	(1.09–1.73)	-	NSCLC	[59]
*TLR4*	rs11536889	OS	1.38	(1.09–3.12)	0.02	Breast cancer	[11]
*TNFRSF10B*	rs11785599	EFS	1.41	(1.16–1.70)	<0.01	NSCLC	[63]
*TNFRSF1B*	rs1061622	OS	0.38	(0.15–0.94)	0.04	NSCLC	[64]
*TNFRSF4*	rs3753348	OS	3.41	(1.65–7.05)	<0.01	Ovarian cancer	[72]

a EFS, event free survival; OS, overall survival; RFS, relapse free survival; DFS, disease free survival; DMFS, distant metastasis-free survival; BCSS, breast cancer specific survival.

b DLBCL, diffuse large B-cell lymphoma; NSCLC, non-small cell lung cancer; FL, follicular lymphoma; HNSCC, head and neck squamous cell carcinoma.

## Discussion

In this study, we found that the rs1952438 in the suppressors of cytokine signaling (*SOCS4)* gene, rs2289278 in the thymic stromal lymphopoietin (*TSLP*) gene and rs2074724 in the hepatocyte growth factor (*HGF*) gene were highly associated with a poor prognosis of breast cancer. Moreover, the polygenic risk score model with genetic variations of immunity-related genes showed that the hazard of DFS of patients was significantly increased as high-risk alleles accumulated. In the GSEA-SNP analysis, 18 pathways significantly affected breast cancer prognosis.

The rs1952438 is located in the intron region of *SOCS4* gene. SOCS family are rapidly induced by activated STATs and negatively regulate JAK/STAT pathway by a classical feedback loop [22]. Furthermore, other signal molecules such as FAK, IRS, p65, GR which are related with carcinogenesis, are regulated by SOCS proteins [23]–[27]. In addition, there are several previous study which reported that people who have higher expression level of *SOCS4* are likely remained disease free status compared to those who developed recurrence [28]. In the view of previous studies which explain functional importance of *SOCS4* and results of present study, it might be assumed that rs1952438 is associated with poorer prognosis of breast cancer by declining expression level of *SOCS4*.

The rs2289278 is found in intron 2 of the long-form of *TSLP* and in the 5′ untranslated region of the short-form of *TSLP* [29]. *TSLP* is a member of the IL-2 cytokine family and a distant paralog of IL-7. *TSLP* may have an important role in tumor progression by activating CD4*+* T cells, inducing the expressing of OX40L in dendritic cells (DCs), and producing Th2-type cytokines and B-cell growth factor [30]. A recent study has shown that breast cancer cells have high expression levels of *TSLP*, indicating that the *TSLP* may be critical in the development of breast cancer [31]. It is that high expression level of TSLP in cancer increases the Th2 level [30]. Furthermore, Th2 cytokines promote disease progres­sion through the increased survival of cancer cells, M2 macrophage differentia­tion, and fibrosis [31], [32]. Thus, TSLP may be an important factor of breast tumor progression and the prognosis of a patient.

The rs2074724 is located in the intron of *HGF*. *HGF* is known to activate angiogenesis of tumors as well as cell-cell interactions, matrix adhesion, migration, invasion [33]. Moreover, breast cancer patients with a high HGF concentration had a significantly poor prognosis when compared to those with a low HGF concentration [34]. Therefore, HGF level was found to be the most important independent factor in predicting the prognosis of breast cancer.

In the GSEA-SNP analysis, there are 18 significant pathways; among these pathways, gene set from Kyng et al [35] which included rs1952438 in *SOCS4* gene and rs2074724, and rs5745752 in *HGF* gene is described that environmental stress such as 4-nitroquinoline-1-oxide (4NQO) elicited DNA damage specific gene expression changes of up to 10. In short, it can be expected that those SNPs included in the pathway can up-regulate breast cancer progression and result in poor prognosis by influencing on environmental response, although there are not precise result in this assumption.

‘Myc tagets1’ gene set from Benporath et al [36] which included rs12458289 and rs9989529 in *BCL2* gene, and rs2142097 in *NBN* gene is shown as the most significant gene set. Benporath et al describe that targets of *Nanog*, *Oct4*, *Sox2* and *c-Myc* are more frequently associated in poorly differentiated tumors than in well-differentiated tumors. *c-Myc* is well known to directly regulate the expression of *NBN* gene involved in DNA double-strand break repair and can result in chromosomal instability, cellular proliferation defects leading to increased more aggressive and metastatic tumor latency [37], [38]. *BCL2* and *c-Myc* are known to make the negative feedback loop in breast cancer cell line [39]. Taking all these consideration of both Benporath et al and results of present study to account, it is can be deduced that rs12458289, rs9989529, and rs2142097 might be associated with the prognosis of breast cancer by interacting with *c-MYC* gene.

To support the indirectly functional effects of our results, we attempted to find potential functional SNPs in *SOCS4*, *HGF*, *TSLP* and genes included in GSEA-SNP using UCSC database [40] and checked the LD between the potential functional SNPs and our findings. Table S2 show the functional SNPs studied in this study and functional SNPs in LD with those SNPs, generally to affect histone modification, DNA methylation, and binding affinity of several transcription factors located in 5′UTR or 3′UTR. For example, transcription activity of *IL-8* is influenced by rs4073 which located in promoter region of *IL-8* [41] and the variant increased the risk of mortality in follicular lymphocytic leukemia by increasing production of *IL-8* [42]. As a result, it is possibly anticipated that those potential SNPs may influence to breast cancer prognosis by regulating the epigenetic and transcriptional pathway.

Several previous reports have evaluated the associations of immunity gene polymorphism and breast cancer prognosis [11]–[14]. They suggested that the variants of *ERCC2, TLR4*, *IL-2*, *IL-6*, and *IL-21* genes had associations with breast cancer prognosis respectively. However, those genes were not replicated in present study. In the other types of cancer studies, *IL-6R*, *IL-8*, *IL-10RB*, *IL-12A*, and *IL-12B* genes were consistently associated with cancer prognosis between our study and theirs. However, there were few consistent SNPs with cancer prognosis in our review of the literature, which may result from various cancer targets, different ethnicities, and different prognostic factors in the models and statistical power.

In this study, there are several limitations including a small sample size and absence of an external validation study. Since the power of this study was low to detect accurate results, the results of this study are carefully interpreted, although the significance levels of top 3 SNPs passed the FDR test with significance (*p<*0.05) and the internal validity was confirmed by the cross-validation. In addition, polygenic risk score model and GSEA-SNP are conducted with whole significant SNPs which include insignificant SNPs at FDR p-value greater than 0.05. Tag SNPs selected based on the data of a Caucasian population and lack of breast cancer subtype information were also limitations of this study. In the systematic review-level, the summary measure and synthesis of the results were not calculated because various genes and the variations related to immune response were the focus. However, the strength of this study is that lots of genetic factors in immune-related genes were covered at once. Moreover, it attempted to apply the candidate gene approach to cover the pathway of immunity-related genetic factors with breast cancer prognosis in Asian women.

In conclusion, our study found that common variants in the *SOCS4*, *TSLP* and *HGF* genes might be related with breast cancer prognosis in Korean women. Hazard of DFS in patients was significantly increased when high-risk alleles were accumulated. Therefore, our results suggest that genetic polymorphisms in immunity-related genes have relevance to breast cancer prognosis among Korean women. Further large-scale functional studies are needed to confirm our findings.

## Supporting Information

Table S1 **PRISMA checklist.**
(DOCX)

Table S2 **Potential functional SNPs which has a LD with SNPs in** ***SOCS4, HGF, TSLP*** **and gene in GSEA-SNP (r^2^>0.8).**
(DOCX)

## References

[1] Jemal A (2012) Global burden of cancer: opportunities for prevention. Lancet 380: 1797–1799. 23079587 10.1016/S0140-6736(12)61688-2

[2] Witz IP, Levy-Nissenbaum O (2006) The tumor microenvironment in the post-PAGET era. Cancer Lett 242: 1–10. 16413116 10.1016/j.canlet.2005.12.005

[3] Jung KW, Won YJ, Kong HJ, Oh CM, Seo HG, et al (2013) Cancer statistics in Korea: incidence, mortality, survival and prevalence in 2010. Cancer Res Treat 45: 1–14. 23613665 10.4143/crt.2013.45.1.1 PMC3629358

[4] Key TJ, Verkasalo PK, Banks E (2001) Epidemiology of breast cancer. Lancet Oncol 2: 133–140. 11902563 10.1016/S1470-2045(00)00254-0

[5] Sariego J, Zrada S, Byrd M, Matsumoto T (1995) Breast cancer in young patients. Am J Surg 170: 243–245. 7661290 10.1016/s0002-9610(05)80007-8

[6] Zhang Y, Cheng S, Zhang M, Zhen L, Pang D, et al (2013) High-infiltration of tumor-associated macrophages predicts unfavorable clinical outcome for node-negative breast cancer. PLoS One 8: e76147. 24098773 10.1371/journal.pone.0076147 PMC3786995

[7] Dan T, Hewitt SM, Ohri N, Ly D, Soule BP, et al (2014) CD44 is prognostic for overall survival in the NCI randomized trial on breast conservation with 25 year follow-up. Breast Cancer Res Treat 143: 11–18. 24276281 10.1007/s10549-013-2758-9 PMC7668685

[8] Mori K, Shibanuma M, Nose K (2004) Invasive potential induced under long-term oxidative stress in mammary epithelial cells. Cancer Res 64: 7464–7472. 15492271 10.1158/0008-5472.CAN-04-1725

[9] Lewis CE, Leek R, Harris A, McGee JO (1995) Cytokine regulation of angiogenesis in breast cancer: the role of tumor-associated macrophages. J Leukoc Biol 57: 747–751. 7539028 10.1002/jlb.57.5.747

[10] Schreibelt G, Tel J, Sliepen KH, Benitez-Ribas D, Figdor CG, et al (2010) Toll-like receptor expression and function in human dendritic cell subsets: implications for dendritic cell-based anti-cancer immunotherapy. Cancer Immunol Immunother 59: 1573–1582. 20204387 10.1007/s00262-010-0833-1 PMC11029854

[11] Yang CX, Li CY, Feng W (2013) Toll-like receptor 4 genetic variants and prognosis of breast cancer. Tissue Antigens 81: 221–226. 23510418 10.1111/tan.12096

[12] You Y, Deng J, Zheng J, Hu M, Li N, et al (2013) IL-21 gene polymorphism is associated with the prognosis of breast cancer in Chinese populations. Breast Cancer Res Treat 137: 893–901. 23288348 10.1007/s10549-012-2401-1

[13] Hu XB, Ouyang LZ, Tang LL (2013) Interleukin-2 gene polymorphisms and prognosis of breast cancer. Genet Test Mol Biomarkers 17: 453–457. 23477313 10.1089/gtmb.2012.0494

[14] DeMichele A, Gray R, Horn M, Chen J, Aplenc R, et al (2009) Host genetic variants in the interleukin-6 promoter predict poor outcome in patients with estrogen receptor-positive, node-positive breast cancer. Cancer Res 69: 4184–4191. 19435922 10.1158/0008-5472.CAN-08-2989 PMC4304767

[15] Lee JY, Park AK, Lee KM, Park SK, Han S, et al (2009) Candidate gene approach evaluates association between innate immunity genes and breast cancer risk in Korean women. Carcinogenesis 30: 1528–1531. 19372141 10.1093/carcin/bgp084

[16] Boyle P, Levin B, Cancer IAfRo (2008) World cancer report 2008: IARC Press.

[17] Park SK, Yang JJ, Oh S, Cho LY, Ma SH, et al (2012) Innate immunity and non-Hodgkin’s lymphoma (NHL) related genes in a nested case-control study for gastric cancer risk. PLoS One 7: e45274. 23028900 10.1371/journal.pone.0045274 PMC3448653

[18] Benjamini Y, Hochberg Y (1995) Controlling the false discovery rate: a practical and powerful approach to multiple testing. Journal of the Royal Statistical Society Series B (Methodological): 289–300.

[19] Reeves GK, Travis RC, Green J, Bull D, Tipper S, et al (2010) Incidence of breast cancer and its subtypes in relation to individual and multiple low-penetrance genetic susceptibility loci. Jama 304: 426–434. 20664043 10.1001/jama.2010.1042

[20] Harrell FE Jr, Lee KL, Mark DB (1996) Multivariable prognostic models: issues in developing models, evaluating assumptions and adequacy, and measuring and reducing errors. Stat Med 15: 361–387. 8668867 10.1002/(SICI)1097-0258(19960229)15:4<361::AID-SIM168>3.0.CO;2-4

[21] Holden M, Deng S, Wojnowski L, Kulle B (2008) GSEA-SNP: applying gene set enrichment analysis to SNP data from genome-wide association studies. Bioinformatics 24: 2784–2785. 18854360 10.1093/bioinformatics/btn516

[22] Hilton DJ (1999) Negative regulators of cytokine signal transduction. Cell Mol Life Sci 55: 1568–1577. 10526574 10.1007/s000180050396 PMC11146996

[23] Emanuelli B, Peraldi P, Filloux C, Chavey C, Freidinger K, et al (2001) SOCS-3 inhibits insulin signaling and is up-regulated in response to tumor necrosis factor-alpha in the adipose tissue of obese mice. J Biol Chem 276: 47944–47949. 11604392 10.1074/jbc.M104602200

[24] Haffner MC, Jurgeit A, Berlato C, Geley S, Parajuli N, et al (2008) Interaction and functional interference of glucocorticoid receptor and SOCS1. J Biol Chem 283: 22089–22096. 18524780 10.1074/jbc.M801041200

[25] Liu E, Cote JF, Vuori K (2003) Negative regulation of FAK signaling by SOCS proteins. Embo j 22: 5036–5046. 14517242 10.1093/emboj/cdg503 PMC204486

[26] Ryo A, Suizu F, Yoshida Y, Perrem K, Liou YC, et al (2003) Regulation of NF-kappaB signaling by Pin1-dependent prolyl isomerization and ubiquitin-mediated proteolysis of p65/RelA. Mol Cell 12: 1413–1426. 14690596 10.1016/s1097-2765(03)00490-8

[27] Ueki K, Kondo T, Kahn CR (2004) Suppressor of cytokine signaling 1 (SOCS-1) and SOCS-3 cause insulin resistance through inhibition of tyrosine phosphorylation of insulin receptor substrate proteins by discrete mechanisms. Mol Cell Biol 24: 5434–5446. 15169905 10.1128/MCB.24.12.5434-5446.2004 PMC419873

[28] Sasi W, Jiang WG, Sharma A, Mokbel K (2010) Higher expression levels of SOCS 1, 3, 4, 7 are associated with earlier tumour stage and better clinical outcome in human breast cancer. BMC Cancer 10: 178. 20433750 10.1186/1471-2407-10-178 PMC2876081

[29] Liu W, Xu LS, Liu QJ, Dong FZ, Qiu RF, et al (2012) Two single nucleotide polymorphisms in TSLP gene are associated with asthma susceptibility in Chinese Han population. Exp Lung Res 38: 375–382. 22913730 10.3109/01902148.2012.714840

[30] Ziegler SF, Roan F, Bell BD, Stoklasek TA, Kitajima M, et al (2013) The biology of thymic stromal lymphopoietin (TSLP). Adv Pharmacol 66: 129–155. 23433457 10.1016/B978-0-12-404717-4.00004-4 PMC4169878

[31] Olkhanud PB, Rochman Y, Bodogai M, Malchinkhuu E, Wejksza K, et al (2011) Thymic stromal lymphopoietin is a key mediator of breast cancer progression. J Immunol 186: 5656–5662. 21490155 10.4049/jimmunol.1100463 PMC3401482

[32] Pedroza-Gonzalez A, Xu K, Wu TC, Aspord C, Tindle S, et al (2011) Thymic stromal lymphopoietin fosters human breast tumor growth by promoting type 2 inflammation. J Exp Med 208: 479–490. 21339324 10.1084/jem.20102131 PMC3058586

[33] Parr C, Watkins G, Mansel RE, Jiang WG (2004) The hepatocyte growth factor regulatory factors in human breast cancer. Clin Cancer Res 10: 202–211. 14734471 10.1158/1078-0432.ccr-0553-3

[34] Yamashita J, Ogawa M, Yamashita S, Nomura K, Kuramoto M, et al (1994) Immunoreactive hepatocyte growth factor is a strong and independent predictor of recurrence and survival in human breast cancer. Cancer Res 54: 1630–1633. 8137271

[35] Kyng KJ, May A, Stevnsner T, Becker KG, Kolvra S, et al (2005) Gene expression responses to DNA damage are altered in human aging and in Werner Syndrome. Oncogene 24: 5026–5042. 15897889 10.1038/sj.onc.1208692

[36] Ben-Porath I, Thomson MW, Carey VJ, Ge R, Bell GW, et al (2008) An embryonic stem cell-like gene expression signature in poorly differentiated aggressive human tumors. Nat Genet 40: 499–507. 18443585 10.1038/ng.127 PMC2912221

[37] Chiang YC, Teng SC, Su YN, Hsieh FJ, Wu KJ (2003) c-Myc directly regulates the transcription of the NBS1 gene involved in DNA double-strand break repair. J Biol Chem 278: 19286–19291. 12637527 10.1074/jbc.M212043200

[38] Shimada M, Sagae R, Kobayashi J, Habu T, Komatsu K (2009) Inactivation of the Nijmegen breakage syndrome gene leads to excess centrosome duplication via the ATR/BRCA1 pathway. Cancer Res 69: 1768–1775. 19244116 10.1158/0008-5472.CAN-08-3016

[39] Gaire RK, Smith L, Humbert P, Bailey J, Stuckey PJ, et al (2013) Discovery and analysis of consistent active sub-networks in cancers. BMC Bioinformatics 14 Suppl 2: S7. 10.1186/1471-2105-14-S2-S7 PMC3549822 23368093

[40] Kent WJ, Sugnet CW, Furey TS, Roskin KM, Pringle TH, et al (2002) The human genome browser at UCSC. Genome Res 12: 996–1006. 12045153 10.1101/gr.229102 PMC186604

[41] Hull J, Thomson A, Kwiatkowski D (2000) Association of respiratory syncytial virus bronchiolitis with the interleukin 8 gene region in UK families. Thorax 55: 1023–1027. 11083887 10.1136/thorax.55.12.1023 PMC1745668

[42] Cerhan JR, Wang S, Maurer MJ, Ansell SM, Geyer SM, et al (2007) Prognostic significance of host immune gene polymorphisms in follicular lymphoma survival. Blood 109: 5439–5446. 17327408 10.1182/blood-2006-11-058040 PMC1890834

[43] Mootha VK, Lindgren CM, Eriksson KF, Subramanian A, Sihag S, et al (2003) PGC-1alpha-responsive genes involved in oxidative phosphorylation are coordinately downregulated in human diabetes. Nat Genet 34: 267–273. 12808457 10.1038/ng1180

[44] Buytaert E, Matroule JY, Durinck S, Close P, Kocanova S, et al (2008) Molecular effectors and modulators of hypericin-mediated cell death in bladder cancer cells. Oncogene 27: 1916–1929. 17952126 10.1038/sj.onc.1210825

[45] Kanehisa M, Goto S, Sato Y, Kawashima M, Furumichi M, et al (2014) Data, information, knowledge and principle: back to metabolism in KEGG. Nucleic Acids Res 42: D199–205. 24214961 10.1093/nar/gkt1076 PMC3965122

[46] Schaefer CF, Anthony K, Krupa S, Buchoff J, Day M, et al (2009) PID: the Pathway Interaction Database. Nucleic Acids Res 37: D674–679. 18832364 10.1093/nar/gkn653 PMC2686461

[47] Ivanova NB, Dimos JT, Schaniel C, Hackney JA, Moore KA, et al (2002) A stem cell molecular signature. Science 298: 601–604. 12228721 10.1126/science.1073823

[48] Zwang Y, Sas-Chen A, Drier Y, Shay T, Avraham R, et al (2011) Two phases of mitogenic signaling unveil roles for p53 and EGR1 in elimination of inconsistent growth signals. Mol Cell 42: 524–535. 21596316 10.1016/j.molcel.2011.04.017 PMC3100487

[49] Nishimura D (2001) BioCarta. Biotech Software & Internet Report: The Computer Software Journal for Scient 2: 117–120.

[50] Ohguchi H, Tanaka T, Uchida A, Magoori K, Kudo H, et al (2008) Hepatocyte nuclear factor 4alpha contributes to thyroid hormone homeostasis by cooperatively regulating the type 1 iodothyronine deiodinase gene with GATA4 and Kruppel-like transcription factor 9. Mol Cell Biol 28: 3917–3931. 18426912 10.1128/MCB.02154-07 PMC2423126

[51] Perez CA, Ott J, Mays DJ, Pietenpol JA (2007) p63 consensus DNA-binding site: identification, analysis and application into a p63MH algorithm. Oncogene 26: 7363–7370. 17563751 10.1038/sj.onc.1210561

[52] Smirnov DA, Foulk BW, Doyle GV, Connelly MC, Terstappen LW, et al (2006) Global gene expression profiling of circulating endothelial cells in patients with metastatic carcinomas. Cancer Res 66: 2918–2922. 16540638 10.1158/0008-5472.CAN-05-4003

[53] Bewick MA, Lafrenie RM, Conlon MSC (2011) Nucleotide excision repair polymorphisms and survival outcome for patients with metastatic breast cancer. Journal of Cancer Research and Clinical Oncology 137: 543–550. 20508946 10.1007/s00432-010-0915-7 PMC11828315

[54] Lu S, Bevier M, Huhn S, Sainz J, Lascorz J, et al (2013) Genetic variants in C-type lectin genes are associated with colorectal cancer susceptibility and clinical outcome. Int J Cancer. 10.1002/ijc.28251 23650115

[55] Castro FA, Forsti A, Buch S, Kalthoff H, Krauss C, et al (2011) TLR-3 polymorphism is an independent prognostic marker for stage II colorectal cancer. Eur J Cancer 47: 1203–1210. 21239167 10.1016/j.ejca.2010.12.011

[56] Slattery ML, Lundgreen A, Bondurant KL, Wolff RK (2011) Interferon-signaling pathway: associations with colon and rectal cancer risk and subsequent survival. Carcinogenesis 32: 1660–1667. 21859832 10.1093/carcin/bgr189 PMC3204348

[57] Bondurant KL, Lundgreen A, Herrick JS, Kadlubar S, Wolff RK, et al (2013) Interleukin genes and associations with colon and rectal cancer risk and overall survival. Int J Cancer 132: 905–915. 22674296 10.1002/ijc.27660 PMC3470814

[58] Bi N, Yang M, Zhang L, Chen X, Ji W, et al (2010) Cyclooxygenase-2 genetic variants are associated with survival in unresectable locally advanced non-small cell lung cancer. Clin Cancer Res 16: 2383–2390. 20332326 10.1158/1078-0432.CCR-09-2793

[59] Dai J, Hu Z, Dong J, Xu L, Pan S, et al (2012) Host immune gene polymorphisms were associated with the prognosis of non-small-cell lung cancer in Chinese. Int J Cancer 130: 671–676. 21412764 10.1002/ijc.26067

[60] Sung WW, Wang YC, Cheng YW, Lee MC, Yeh KT, et al (2011) A polymorphic −844T/C in FasL promoter predicts survival and relapse in non-small cell lung cancer. Clin Cancer Res 17: 5991–5999. 21807637 10.1158/1078-0432.CCR-11-0227

[61] Yuan X, Wei Q, Komaki R, Liu Z, Yang J, et al (2013) TGF(beta)1 Polymorphisms Predict Distant Metastasis-Free Survival in Patients with Inoperable Non-Small-Cell Lung Cancer after Definitive Radiotherapy. PLoS ONE 8. 10.1371/journal.pone.0065659 PMC3686751 23840350

[62] Xue SL, Zheng YH, Su HF, Deng X, Zhang XB, et al (2013) Association between single nucleotide polymorphisms of the transforming growth factor-beta1 gene and overall survival in unresectable locally advanced non-small-cell lung cancer patients treated with radio(chemo)therapy in a Chinese population. Medical Oncology 30. 10.1007/s12032-013-0512-0 23435990

[63] Schabath MB, Giuliano AR, Thompson ZJ, Amankwah EK, Gray JE, et al (2013) TNFRSF10B polymorphisms and haplotypes associated with increased risk of death in non-small cell lung cancer. Carcinogenesis 34: 2525–2530. 23839018 10.1093/carcin/bgt244 PMC3810840

[64] Guan X, Liao Z, Ma H, Qian J, Liu Z, et al (2011) TNFRSF1B +676 T>G polymorphism predicts survival of non-Small cell lung cancer patients treated with chemoradiotherapy. BMC Cancer 11. 10.1186/1471-2407-11-447 PMC3220654 21995493

[65] Pine SR, Mechanic LE, Ambs S, Bowman ED, Chanock SJ, et al (2007) Lung cancer survival and functional polymorphisms in MBL2, an innate-immunity gene. Journal of the National Cancer Institute 99: 1401–1409. 17848669 10.1093/jnci/djm128 PMC6278934

[66] Guirado M, Gil H, Saenz-Lopez P, Reinboth J, Garrido F, et al (2012) Association between C13ORF31, NOD2, RIPK2 and TLR10 polymorphisms and urothelial bladder cancer. Hum Immunol 73: 668–672. 22504414 10.1016/j.humimm.2012.03.006

[67] Schutz FA, Pomerantz MM, Gray KP, Atkins MB, Rosenberg JE, et al (2013) Single nucleotide polymorphisms and risk of recurrence of renal-cell carcinoma: a cohort study. Lancet Oncol 14: 81–87. 23219378 10.1016/S1470-2045(12)70517-X PMC3769687

[68] Aschebrook-Kilfoy B, Zheng T, Foss F, Ma S, Han X, et al (2012) Polymorphisms in immune function genes and non-Hodgkin lymphoma survival. J Cancer Surviv 6: 102–114. 22113576 10.1007/s11764-010-0164-4 PMC3326600

[69] Charbonneau B, Maurer MJ, Fredericksen ZS, Zent CS, Link BK, et al (2012) Germline variation in complement genes and event-free survival in follicular and diffuse large B-cell lymphoma. Am J Hematol 87: 880–885. 22718493 10.1002/ajh.23273 PMC3586263

[70] Habermann TM, Wang SS, Maurer MJ, Morton LM, Lynch CF, et al (2008) Host immune gene polymorphisms in combination with clinical and demographic factors predict late survival in diffuse large B-cell lymphoma patients in the pre-rituximab era. Blood 112: 2694–2702. 18633131 10.1182/blood-2007-09-111658 PMC2556607

[71] Lenci RE, Bevier M, Brandt A, Bermejo JL, Sucker A, et al (2012) Influence of genetic variants in type I interferon genes on melanoma survival and therapy. PLoS One 7: e50692. 23209811 10.1371/journal.pone.0050692 PMC3507747

[72] Goode EL, DeRycke M, Kalli KR, Oberg AL, Cunningham JM, et al (2013) Inherited variants in regulatory T cell genes and outcome of ovarian cancer. PLoS One 8: e53903. 23382860 10.1371/journal.pone.0053903 PMC3559692

[73] Reid-Lombardo KM, Fridley BL, Bamlet WR, Cunningham JM, Sarr MG, et al (2013) Survival is associated with genetic variation in inflammatory pathway genes among patients with resected and unresected pancreatic cancer. Annals of Surgery 257: 1096–1102. 23360921 10.1097/SLA.0b013e318275b7e5 PMC3677709

[74] Biason P, Hattinger CM, Innocenti F, Talamini R, Alberghini M, et al (2012) Nucleotide excision repair gene variants and association with survival in osteosarcoma patients treated with neoadjuvant chemotherapy. Pharmacogenomics Journal 12: 476–483. 21826087 10.1038/tpj.2011.33 PMC3935514

[75] Lee JM, Yang PW, Yang SY, Chuang TH, Tung EC, et al (2011) Genetic variants in DNA repair predicts the survival of patients with esophageal cancer. Annals of Surgery 253: 918–927. 21490450 10.1097/SLA.0b013e318216f374

[76] Lundberg M, Saarilahti K, Makitie AA, Mattila PS (2010) TGF(beta)1 genetic polymorphism is associated with survival in head and neck squamous cell carcinoma independent of the severity of chemoradiotherapy induced mucositis. Oral Oncology 46: 369–372. 20308003 10.1016/j.oraloncology.2010.02.012

[77] Vangsted AJ, Klausen TW, Ruminski W, Gimsing P, Andersen NF, et al (2009) The polymorphism IL-1(beta) T-31C is associated with a longer overall survival in patients with multiple myeloma undergoing auto-SCT. Bone Marrow Transplantation 43: 539–545. 18997828 10.1038/bmt.2008.351

