# Supplementary material for: The Associations between Immunity-Related Genes and Breast Cancer Prognosis in Korean Women
Source: PLoS One. 2014 Jul 30;9(7):e103593. doi: 10.1371/journal.pone.0103593 (PMC4116221; doi:10.1371/journal.pone.0103593)
Supplement: Table S2 — Potential functional SNPs which has a LD with SNPs in SOCS4, HGF, TSLP and gene in GSEA-SNP (r2>0.8). (DOCX) [file pone.0103593.s002.docx]

Table S2 Potential functional SNPs which has a LD with SNPs in *SOCS4, HGF, TSLP* and gene in GSEA-SNP (r^2^ > 0.8)

| Gene | SNP^1^ | SNP^2^ | Distance | r^2^ | location | Function^3^ |
| --- | --- | --- | --- | --- | --- | --- |
| *SOCS4* | rs1952438 |  |  |  | intron | Histone modification |
|  |  | rs77911976 | 3857 | 1.000 | 5’ UTR | Histone modification  DNA methylation  >40 TFBS |
| *HGF* | rs2074724 |  |  |  | intron | - |
|  |  | rs4732402 | 5885 | 0.908 | 3’ UTR | Histone modification |
| *ALOXE3* | rs3027215 |  |  |  | intron | - |
|  |  | rs3027229 | 3102 | 0.867 | 5’ UTR, missense | Histone modification  DNA methylation  >5 TFBS |
| *IL-4R* | rs8832 |  |  |  | 3’ UTR | Histone modification  4 TFBS |
|  |  | rs1029489 | 430 | 1.000 | 3’ UTR | 8 TFBS |
| *IL-8* | rs4694178 |  |  |  | intergenic | - |
|  |  | rs13112910 | 2933 | 0.963 | 3’ UTR | Histone modification  TFBS (POLR2A) |
|  |  | rs4073 | 6664 | 0.963 | 5’ UTR | Histone modification  7 TFBS |
|  |  | rs1126647 | 3643 | 0.851 | 3’ UTR | Poly A site  Histone modification |
|  |  | rs10938092 | 2973 | 0.815 | 3’UTR | Histone modification  TFBS (POLR2A) |
| *IRF4* | rs11242867 |  |  |  | intergenic | Histone modification |
|  |  | rs1050975 | 7394 | 0.898 | 3’ UTR | ncRNA_exonic  Histone modification |
| *KLK15* | rs3745523 |  |  |  | upstream | - |
|  |  | rs266855 | 3494 | 0.918 | 5’ UTR | Histone modification  DNA methylation |

^1^SNPs which studied in this study

^2^Potential functional SNPs which had a LD with results of our study

^3^TFBS, transcription factor binding site
